# Dr. L. Martinet's Manual of Pathology

**Published:** 1827-04

**Authors:** 


*Manual of Pathology; containing the Symptoms, Diagnosis, and Morbid Characters of Diseases: together with an Exposition of the different Methods of Examination, applicable to Affections of the Head, Chest, and Abdomen.* By L. MARTINET, D. M. P. Resident Physician of the Hôtel Dieu. Translated, with Notes and Additions, by JONES QUAIN, A.B. Demonstrator of Anatomy at the Medical School, Aldersgate Street. Duodecimo, p. 310. Anderson, October, 1826.

WE need not say that the knowledge of anatomy is necessary for every practitioner; and that the SURGEON ought to have a very minute acquaintance with the human structure, before he dares to operate or undertake the cure of surgical diseases. The physician should have a very clear knowledge of all parts of the body, and more especially of all the internal viscera, in order to be able to ascertain the seats and symptoms of diseases —and the general practitioner, uniting in his own person, the character of both physician and surgeon, should surely be well versed in the structure of that body on which he is operating medicinally and chirurgically from morning till night—and sometimes from night till morning. All this is granted on every side. But the student thinks if he can demonstrate and enumerate every artery, nerve, muscle, and viscus in the human frame, he is a very clever fellow, and prepared for all contingencies; yet, were it not that morbid structure cannot be learnt without a knowledge of sound structure, we would be almost inclined to say that the *former* is the more important and useful study, at least for the physician and the general practitioner. How frequently do we see the most interesting specimens of pathology (meaning the effects of disorders, or morbid alterations) passed over as either misunderstood, or misconstrued by the practitioner who examines the body of a patient! How many false conclusions are drawn over the dead body, for want of a knowledge of morbid anatomy! The knowledge of simple anatomy, without the knowledge of morbid structure, is like the knowledge of physiology, or the laws of health, without any study of diseases. What sort of a practitioner would the most eminent physiologist make, who never attended to the effects of disease in modifying or reversing the laws of healthy function? Yet in such predicament is the student who goes away from his lectures, and classes without an acquaintance with the appearances presented in the dead body after diseases.

Nor will a bare catalogue of the morbid appearances presented in the dead body, on dissection, be of much use to the student, unless he be acquainted with the previous history of the case, and is able to connect cause and effect together. In this respect the original work of Dr. Baillie was remarkably defective. It is evident that his Morbid Anatomy was nothing more than the sepulchretum of the anatomical theatre in Windmill-street, and that he knew nothing of the histories of those diseases which had wrought the changes he so faithfully described. The *symptoms* which he *afterwards* appended, were, unquestionably *afterwards observed*, and are rather the symptoms of diseases in general than those which certainly appeared where the disorganizations enumerated were going on.

We are the more inclined to hazard this remark, because the symptoms during life, and the morbid phenomena in the dead body were but seldom connected at the time Dr. Baillie wrote, either in this country or on the continent—and still more rarely in the high practice of Dr. Baillie, than in the humble spheres of life. How far Dr. Baillie might have availed himself of the facilities afforded by St. George's Hospital, in perfecting his work, we have no means of knowing; but one thing is certain, that the symptoms were not drawn from the cases which afforded the materials of the plates or letter-press of his great work.

Independent of all these considerations, the immense progress which has been made in pathology during the last 20 years, rendered a new work on this important branch absolutely necessary; and, as it is universally allowed that the French have made greater advances in this department than ourselves—partly from greater opportunities and facilities in their hospitals—and partly, we are sorry to observe, from their greater zeal, Mr. Quain has done a service to his countrymen in translating the Manual of Pathology drawn up by Dr. Martinet—the best, and indeed the only work which is fit for the dissecting room in this country. We have no hesitation in asserting, that this manual is equally as necessary as any of those manuals of anatomy which the student employs in his anatomical pursuits, and far more beneficial afterwards, in private practice, when he has opportunities of comparing symptoms with dissections. On this account, the physician, surgeon, and general practitioner should make the work a book of constant reference whenever they proceed to a post mortem examination. It is only necessary, after what we have said, to give a single specimen of the publication, and then we shall leave our brethren to decide for themselves.

### ENCEPHALITIS.

"413. Inflammation of the brain may occur at any period of life from infancy to old age. There are usually some premonitory symptoms, such as a sense of weight in the head, of tinglings in the ears, deception of vision, irritability of the retina, numbness of one side of the body, pain or prickling of the limbs; when suddenly there supervenes a state of contraction or convulsion, continued or intermittent, of the muscles of one side of the body, or only of one of its regions. If the intellectual faculties be not altogether destroyed, the patient complains of headache usually referred to the side opposite to that which is the seat of the contractions; there is no delirium, the understanding is not deranged, it is merely weakened. Sometimes the contracted limbs are painful, particularly when they are flexed, and an effort is made to extend them; the pupil of the affected side is in some instances contracted, and the eye closed by the contraction of the orbicularis muscle; the commissure of the lips is drawn outwards even when the mouth is not moved; but when any voluntary motions are made, the commissure of the opposite side experiences a deviation; the muscles of the neck are in a state of rigidity, and draw the head towards the affected side. Still these various effects of irritation diminish gradually in intensity, and are succeeded by symptoms of collapse; the muscles fall into a state of paralysis with flaccidity; the eye remains closed, but it is by relaxation; the commissure of the lips hitherto contracted becomes pendent; the head and mouth are drawn in the direction *opposite* to that to which they had previously inclined; that is to say, to the sound side; the pupil is dilated, the sensibility of the affected side totally lost, and the understanding completely destroyed. We may here remark, that in order to trace these different effects of the disease, we must observe the patient from the first invasion of the attack to its final termination.

"414. In some cases, we find that a rigid state of the muscles supervenes after a sudden paralysis with flaccidity; this is caused by the apoplexy being followed by encephalitis; the walls of the cavity, in which the effusion had taken place, being then seized with inflammation.

"415. If convulsions attack the side that remained unaffected, and if they be not followed by paralysis, they are caused by the occurrence of inflammation of the arachnoid membrane. If, however, a paralysis succeeds, it arises from a new inflammation attacking the opposite side.

"416. And finally, when encephalitis succeeds to arachnitis, particularly of the base of the brain, as occurs usually in children, one of the sides affected by convulsious becomes paralyzed.

"417. Encephalitis presents several groups of symptoms, each indicating a lesion of a particular part of the brain. Affections of the upper extremity seem referable to lesions of the posterior fibres of the optic thalamus of the opposite side; those of the lower extremity to alterations of the anterior half of the corpus striatum.

"418. Paralysis of both sides of the body at the same time depends on an alteration of the central part of the pons Varolii.

"419. When there is no paralysis or muscular rigidity at either side of the body, and when a comatose state occurs, and goes on progressively increasing, we may suspect inflammation of the corpus callosum, septum lucidum, or fornix.

"420. Loss of the power of utterance seems to depend on an alteration of the anterior lobules of the hemispheres.

"421. Strabismus, rotation of the eye, dilation, contraction, immobility, constant oscillation of the pupil at one side, indicate usually an alteration of the surface of the corpora quadrigemina of the opposite side.

"422. Lesions of the pituitary gland, of the infundibulum, and of the grey lamella in which it terminates, by causing compression of the optic nerve at one side behind the point of decussation, may induce blindness of the opposite eye.

" 423. As to alterations of the transparency of the membranes and humours of the eye, and to paralysis of the organs of sense at one side, they seem to depend either on a derangement of the ganglion of the fifth pair of nerves where it lies on the petrous portion of the temporal bone, or a lesion of the corresponding walls of the fourth ventricle.

" 424. Finally, derangements of the circulation, respiration, and of the generative system, without paralysis of the limbs, indicate an alteration of one of the lobes of the cerebellum.

" 425. *The diseases with which it may be confounded* are, hæmorrhage, or 'ramollissement' of the substance of the brain, nervous fever, some cases of arachnitis, especially when it is circumscribed, and local effusions.

" 426. *Anatomical Characters*,—The inflamed part of the brain presents different appearances, according to the time that the disease has lasted. When it is only of some days duration, the white substance, and, still more perceptibly, the grey exhibits a rosy or slightly red colour, and in it we perceive several vascular filaments. The firmness of the affected part is considerably diminished, and when cut into, the surface of the incision presents (not a multitude of minute drops of blood re-appearing after being wiped away, as occurs in congestion, but) a multitude of small red points, which cannot be removed by ablution. We frequently have occasion to observe these appearances in the cortical substance of the convolutions after arachnitis or violent congestions of the pia mater. In a more advanced stage of encephalitis the brain is red, the vascular injection more strongly marked, and the 'ramollissement' very considerable. Finally, in some cases the blood becomes so intimately combined with the cerebral substance, that its colour approaches that of the lees of wine, being of a deep, dusky red; there is no actual effusion of blood, except we consider as such some small dots about the size of a pin's head, which we occasionally find in some particular points; in such cases the brain is in a state of extreme 'ramollissement, or softening.'

" 427. If it should happen that the inflammation proceeds to these two latter stages without causing death, then the part affected begins gradually to lose its softness, and ultimately becomes more dense than in the natural state; it retains for some time its red colour, but changes finally to a dusky yellow.

" 428. The third stage of encephalitis is that of suppuration; the red colour gradually disappears, the blood is replaced by a sero-purulent fluid, which is infiltered into the substance of the brain, combines with it, and gives to it, according to the extent of the admixture, a greyish dull white, or yellowish green colour. The pus accumulates in some spots to a greater or less extent; sometimes there are no more than one or two drops, but still they are easily recognized by their resemblance to the pus of ordinary phlegmonia; in other cases, however, it occupies the entire of the centre of one hemisphere where, extravasated as it were, it forms cavities for itself, in which we find mixed with it several fragments of cerebral substance; lastly, in some cases, we find several small cavities uniting together to form a large one.

"429. These cavities are sometimes found separated from the substance of the brain by a new membrane, formed of the remains of the cellular tissue and vessels, which had escaped the effects of the suppuration, and which, when compressed towards the circumference of the cavity, interlace mutually, become organized, gradually increase, and become changed into a membrane whose thickness and density are progressively augmented. The internal surface of these cysts becomes smooth; the pus which they contain assumes more and more the characters of pus formed in cellular tissue, by reason of the progressive destruction of the cerebral substance, and finally becomes white, yellowish, or greenish, and perfectly homogeneous. Sometimes when the abscess is seated near the convolutions, the pia mater and arachnoid becoming thickened, concur in the formation of its walls. The pus of abscesses in the brain rarely emits any odour, except such as occurs in consequence of caries of the bones of the head, particularly of the petrous portion of the temporal bone; in which cases it is always fetid, and the membranes are altered and perforated.

"430. The grey substance is the most usual seat of encephalitis; and the parts most commonly affected are the corpora striata, optic thalami, the convolutions, pons Varolii, and cerebellum." 142.

From this specimen, which has been taken at random, and which is really one of the least favourable, our readers will perceive that the work actually presents no bad system of symptomatology, in addition to the important department of pathology. The work is printed with all possible regard to convenience in size and economy, and is highly deserving of the patronage of the British public.

